# Cultural Differences in the Hedonic Rewards of Recalling Kindness: Priming Cultural Identity with Language

**DOI:** 10.1007/s42761-020-00029-3

**Published:** 2021-03-23

**Authors:** Lilian J. Shin, Seth M. Margolis, Lisa C. Walsh, Sylvia Y. C. L. Kwok, Xiaodong Yue, Chi-Keung Chan, Nicolson Yat-Fan Siu, Kennon M. Sheldon, Sonja Lyubomirsky

**Affiliations:** 1 Department of Psychology, University of California, Riverside, Riverside, CA USA; 2 Department of Health Disparities Research, University of Texas M.D. Anderson Cancer Center, 1400 Pressler Street, Unit 1440, Houston, TX 77030 USA; 3 Department of Social and Behavioural Sciences, City University of Hong Kong, Hong Kong, Hong Kong; 4 School of Psychology, Capital Normal University, Beijing, China; 5 Department of Counselling and Psychology, Hong Kong Shue Yan University, Hong Kong, Hong Kong; 6 Department of Psychological Sciences, University of Missouri, Columbia, MO USA; 7 Higher School of Economics, National Research University, Moscow, Russia

**Keywords:** Emotion, Well-being, Culture, Kind acts, Priming

## Abstract

Recent theory suggests that members of interdependent (collectivist) cultures prioritize in-group happiness, whereas members of independent (individualist) cultures prioritize personal happiness (Uchida et al. *Journal of Happiness Studies*, *5*(3), 223–239 Uchida et al., 2004). Thus, the well-being of friends and family may contribute more to the emotional experience of individuals with collectivist rather than individualist identities. We tested this hypothesis by asking participants to recall a kind act they had done to benefit either close others (e.g., family members) or distant others (e.g., strangers). Study 1 primed collectivist and individualist cultural identities by asking bicultural undergraduates (*N* = 357) from Hong Kong to recall kindnesses towards close versus distant others in both English and Chinese, while Study 2 compared university students in the USA (*n* = 106) and Hong Kong (*n* = 93). In Study 1, after being primed with the Chinese language (but not after being primed with English), participants reported significantly improved affect valence after recalling kind acts towards friends and family than after recalling kind acts towards strangers. Extending this result, in Study 2, respondents from Hong Kong (but not the USA) who recalled kind acts towards friends and family showed higher positive affect than those who recalled kind acts towards strangers. These findings suggest that people with collectivist cultural identities may have relatively more positive and less negative emotional experiences when they focus on prosocial interactions with close rather than weak ties.

**Supplementary Information:**

The online version contains supplementary material available at 10.1007/s42761-020-00029-3.

Because most people around the globe want to be happy (Diener, 2000; Veenhoven, 2012), empirically supported approaches to improving well-being—in both happy and less happy nations—have generated great interest (e.g., Lyubomirsky, 2008). Over the past two decades, researchers have been testing whether simple, self-administered cognitive and behavioral strategies—known as positive activity interventions (PAIs)—can increase happiness and positive emotion (Seligman et al., 2005; Sheldon et al., 2012; Sin & Lyubomirsky, 2009). One positive activity that has been robustly shown to boost well-being in Western cultures is performing acts of kindness (Dunn et al., 2008; Layous, Nelson, & Lyubomirsky, 2013; Layous, Lee, Choi, & Lyubomirsky, 2013; Lyubomirsky et al., 2005; Nelson et al., 2015, 2016; Sheldon et al., 2012). Kind (or prosocial) behaviors are intentional acts undertaken to benefit others, regardless of the underlying motives, and can include behaviors such as giving a compliment, paying for another’s meal, or helping a colleague with a work task. Research has demonstrated that people who engage in prosocial behavior spontaneously or after being prompted experimentally report increases in well-being (Chancellor et al., 2018; Dunn et al., 2008; Meier & Stutzer, 2008; Schwartz et al., 2003; Sheldon et al., 2012). Interestingly, merely *recalling* or reporting prior acts of kindness by listing or describing what one did can be as effective at increasing well-being as actually performing them (Ko et al., 2019; cf. Otake et al., 2006).

An important unsettled question, however, concerns whether engaging in prosocial behavior is equally hedonically rewarding in collectivist cultures (characterized by interdependent self-construals) and individualist cultures (characterized by independent self-construals; Triandis & Gelfand, 1998). Markus and Kitayama (1991) define an independent self-construal as a view of the self in which individuals see themselves as autonomous entities who assert their rights and act agentically. In contrast, the Eastern, interdependent self-construal is defined as a view of oneself as connected, relational, and concerned about harmonious belonging to a larger collective group.

Applying the concept of independent self-construal to the pursuit of happiness, independent subjective well-being is generally characterized by an explicit striving for personal happiness that may involve mastering one’s environment and achieving goals (including social goals) independently (Uchida et al., 2004). In other words, with an independent approach to pursuing subjective well-being, the ultimate goal is personal happiness, even if this pursuit involves other people. Therefore, subjective well-being in individualist cultures such as the USA encompasses being kind to both one’s in-group(s) and out-group(s), as long as the end result is individual (personal) happiness (Triandis, 2001).

Interdependent subjective well-being, on the other hand, emphasizes taking one’s proper place, perfecting one’s roles, empathizing with others, acting on the bases of others’ expectations, and blurring the distinction between self and others (Hitokoto & Uchida, 2015; Uchida et al., 2004)—that is, the ultimate goal is the well-being of the in-group through harmonious and fulfilling relationships rather than one’s distinct personal happiness (Triandis, 2001). Accordingly, relative to individualist cultures, we argue that there is a clearer distinction in collectivist cultures (e.g., East Asian cultures) between one’s in-group(s) and out-group(s), and the well-being of the in-group plays a relatively larger role in one’s personal happiness.

However, PAIs have rarely been specifically designed with interdependent self-construals in mind, and few empirical studies have compared their efficacy in Asian (collectivist) versus European or American (individualist) participants (for exceptions, see Layous, Nelson, & Lyubomirsky 2013; Layous, Lee, Choi, & Lyubomirsky 2013; Shin, Walsh, & Lyubomirsky 2019; Shin, Layous, Choi, Na, & Lyubomirsky, 2019). To address this gap, we used two different approaches to test the efficacy of a happiness intervention—“recalling kindness” (Otake et al., 2006) towards in-group vs. out-group members—in collectivist and individualist cultures and identities.

## Present Research and Hypotheses

In Study 1, we primed collectivist (Asian) versus individualist (European) cultural identity in bicultural individuals from Hong Kong. Due to Hong Kong’s historical-political context, many individuals in Hong Kong identify as bicultural, associating with both Eastern and Western cultures (Ng & Lai, 2011). Importantly, cultural practices and meanings appear to be deeply embedded in language, and, as a result, bicultural individuals may possess separate self-structures associated with different languages (Ross et al., 2002; Wang et al., 2010). Research has shown that it is possible to prime one cultural identity over the other in bicultural individuals through imagery or language (Hong et al., 2000; Menon et al., 2010; Ramírez-Esparza et al., 2006). To this end, our study manipulated primed cultural identity via Chinese versus English language within participants and examined hedonic differences after recalling kindnesses in these two languages. We hypothesized that when recalling acts of kindness in Chinese, kindnesses towards close others would elicit a more positive affective experience than kindnesses towards distant others. By contrast, when recalling kind acts in English, the nature of the targets (i.e., close vs. distant others) would not impact affect ratings. These hypotheses were pre-registered on the Open Science Framework (https://tinyurl.com/y9ld6du4); data, code, and materials can also be found on this website.

In Study 2, a between-subjects rather than a within-subjects design was used, in which we recruited participants from different cultural groups to determine whether any effects of target found in Study 1 were unique to bilingual participants in Hong Kong. In this study, participants in the USA and Hong Kong recalled a kindness they had done that benefitted either close others or distant others. Parallel to Study 1, we hypothesized that respondents from Hong Kong who recalled kind acts towards friends and family would report higher positive affect than those who recalled kind acts towards strangers. By contrast, we expected that US participants who recalled kind acts towards strangers would show similar levels of positive affect as those who recalled kind acts towards close others.

## Study 1

Using a cultural priming paradigm, Study 1 aimed to test the hedonic effects of recalling a kindness within bicultural individuals from Hong Kong to determine whether the effect of the target of the recalled kindness (close others vs. strangers) depended on the language primed (English vs. Chinese).

### Method

#### Design

This study used a mixed 2 × 2 × 2 design **(**pre-registered) with two within-subject language primes (Chinese vs. English), two between-subject targets of kindness prompts (close other vs. stranger), and two language prime orders (English-then-Chinese vs. Chinese-then-English, counterbalanced). We aimed to recruit 75–100 participants per condition, as an *n* of 100 per condition would allow us 80% power to detect an effect size of *r* = 0.2; 100 is the generally recommended sample size per cell (Vazire, 2014). After removing (1) duplicate cases and (2) cases that responded identically to 12 consecutive items (as pre-registered), the final sample sizes for each condition and time point were as follows: Stranger/Chinese-then-English (T_1_: *n* = 89, T_2_: *n* = 86), Close Other/Chinese-then-English (T_1_: *n* = 94, T_2_: *n* = 86), Stranger/English-then Chinese (T_1_: *n* = 82, T_2_: *n* = 81), and Close Other/English-then-Chinese (T_1_: *n* = 92, T_2_: *n* = 91).

#### Participants

Undergraduates (*N* = 357) from both private (*n* = 178) and public research universities (*n* = 179) in Hong Kong participated in this study. Participants (*M*_age_ = 21.03; range = 17–45 years) were Asian (100%) and predominantly female (79.8%). All were born in either Hong Kong (79%) or Mainland China (21%), with the vast majority growing-up in Hong Kong (92.7%). Participants were eligible to join the study if they were able to read and write both in English and in Traditional or Simplified Chinese. Students either received school credit in exchange for their participation or participated on a voluntary basis, with the private university offering a coupon book or raffle prize as an extra incentive.

#### Procedure

All of the following procedures were pre-registered. Students were recruited through Hong Kong university email lists or individual classes. The two time point study was conducted entirely online, with one time point completed on Day 1 (T_1_) and the second on Day 8 (T_2_). At both time points, students logged in to a Qualtrics survey to receive writing activity instructions and complete measures.

Prior to beginning the study, all participants were randomly assigned to one of four groups, all of which involved recalling a kindness. Specifically, they were asked to recall and write about a time that they were kind to (1) a stranger, first in English (at T_1_) and then in Chinese (at T_2_) (Stranger in English-then-Chinese condition); (2) a stranger, first in Chinese (at T_1_) and then in English (at T_2_) (Stranger in Chinese-then-English condition); (3) a close other, first in English (at T_1_) and then in Chinese (at T_2_) (Close Other in English-then-Chinese condition); or (4) a close other, first in Chinese (at T_1_) and then in English (T_2_) (Close Other in Chinese-then-English condition).

When participants were asked to recall and write about kind acts in English, they completed a survey that was written entirely in English, including all instructions and measures. When participants were asked to recall and write about kind acts in Chinese, they completed a survey that was written entirely in Chinese (Traditional or Simplified based on participants’ Chinese language preferences), including all instructions and measures.

First, at T_1_, participants were sent a survey link in their assigned language (English or Chinese). After consenting, they completed some demographic questions (e.g., age, sex) and moderator measures (e.g., personality, cultural identity; see Supplemental Material). Following these measures, participants completed their assigned recalling kindness writing activity (stranger or close other) and then completed a series of outcome measures.

Seven days later, participants were sent a second online survey in their assigned language (English or Chinese). As part of our cover story, the second survey included a welcome note asking participants to answer all questions—even if they had seen them previously—ostensibly because data would be sent to both Chinese-speaking and English-speaking universities. Participants then completed a T_2_ survey that was identical to the T_1_ survey, except in a different language. Upon completing the second survey, participants received a debriefing statement explaining the study.

##### Recalling Kindness Writing Activity

Participants completed a writing activity about recalling kindnesses either towards a close other or stranger in either English or Chinese, depending on their condition. The close other prompt read as follows:

Please take a moment to reflect upon the past few months. We would like you to try to remember an instance in which you have done something to contribute to the well-being or success of a close other. This person can be a family member/relative, close friend, roommate, close classmate, or close colleague/co-worker. For the next 5-10 minutes, write about what act or acts you have done to benefit or help this individual and reflect upon how your action(s) affected this individual as well as yourself…. Describe in specific terms the kind act or acts you performed and how it affected the person’s life as well as your own life.

For the stranger condition, the prompt remained identical, with the exception of the third line, which read as follows: “…to the well-being or success of a stranger. This person can be anyone whom you do not know—e.g., a grocery clerk, student on campus, or member of your gym.”

#### Measures

All English measures and instructions were translated into Simplified and Traditional Chinese in advance by Chinese-English bilinguals, and then checked and updated for accuracy by multiple collaborators in Hong Kong.

##### Demographic Information

We asked participants general demographic information, such as age, sex, and parents’ education level. We also asked them where they were born and grew-up and what language(s) they speak (English, Chinese [Cantonese], Chinese [Mandarin], or other).

##### Affective Well-Being Outcome Measure

Immediately after the writing activity, we administered a modified Affect-Adjective Scale (AAS; Diener & Emmons, 1985) to assess the extent to which participants felt positive and negative emotions following the recalling kindness activity. This 12-item measure assesses a range of positive emotions (happy, pleased, joyful, enjoyment/fun, peaceful/serene, relaxed/calm) and negative emotions (worried/anxious, angry/hostile, frustrated, depressed/blue, unhappy, dull/bored). To incorporate low arousal emotions found to be characteristic of Asian individuals (Tsai & Park, 2014), we included “peaceful/serene” and “relaxed/calm” among the original set of four positive emotions and “dull/bored” among the original set of five negative emotions in the AAS. Affect valence was computed by subtracting the mean score of all negative emotions from the mean score of all positive emotions. Participants rated the extent to which they were feeling the emotions right now (1, *not at all*; 7, *extremely*). This measure was administered at both T_1_ and T_2_. Scale reliabilities (McDonald’s omegas) for positive affect, negative affect, and affect valence were all 0.88 across both time points. Other outcomes and moderators collected for a different purpose are listed in Supplemental Material.

### Results

#### Manipulation Check

First, to check that our language priming manipulation was successful, we asked participants, “From 0 to 100 percent (total should sum to 100%), to what extent do you feel “Eastern/Asian/Chinese,” “Western/European/American,” or “like you belong to another cultural group that is not Eastern/Asian/Chinese or Western/European/American.” We found a significant difference, such that respondents indicated that they felt more “Western/European/American” when asked in English (collapsed over Time 1 and Time 2; *M* = 13.38, *SD* = 18.35) than when asked in Chinese (*M* = 9.03, *SD* = 14.96, *b* = 3.96, 95% CI [2.49, 5.43], *p* < 0.0001).

Furthermore, to check whether participants recalled comparable kind acts (regarding closeness to target and size of the act) in each language, two Chinese-English bilingual raters independently coded the participants’ written descriptions of their recalled kind acts on two questions: “How close is the author to the person for whom they did an act of kindness? (1, stranger; 2, acquaintance; 3, casual friend or casual colleague; 4, friend or family member; 5, very close friend/colleague [e.g., best friend] or family member [e.g., spouse]) and “How big was the kind act?” (1, small [e.g., holding door or saying thank you]; 2, medium [e.g., gave pocket change to homeless]; 3, big [e.g., spend weekends helping at homeless shelter]).

Cohen’s kappas for these two questions were excellent, at 0.94 and 0.82, respectively. Both coding questions were analyzed with a 2 (Target: Stranger vs. Close Other) × 2 (Language: Chinese vs. English) ANOVA. Mean values for the closeness-to-target coding were as follows: Stranger-Chinese *M* = 1.50, Close Other-Chinese *M* = 3.72, Stranger-English *M* = 1.61, and Close Other-English *M* = 3.97. Mean values for the size-of-act coding were as follows: Stranger-Chinese *M* = 1.56, Close Other-Chinese *M* = 2.45, Stranger-English *M* = 1.90, and Close Other-English *M* = 2.47.

These codings revealed that our participants chose to recall kind acts towards people closer to them (in both the stranger and close other conditions) when reporting in English than when reporting in Chinese, *F* (1715) = 5.54, *p* = 0.02. Additionally, as expected, those who were asked to recall kind acts towards friends and family were coded as reporting kind acts towards people closer to them than those who were asked to recall kind acts towards strangers, *F* (1715) = 889.58, *p* < 0.001. Importantly, however, the interaction was not significant, *F* (1715) = 0.84, *p* = 0.36; participants did not write about targets closer to them in the close other (versus distant other) condition when recalling in Chinese than when recalling in English.

Furthermore, when recalling in English, participants reported bigger acts than when recalling in Chinese, *F* (1715) = 15.46, *p* < 0.001. Additionally, not surprisingly, our codings revealed that those who were asked to recall kind acts towards close others recalled bigger acts than those who were asked to recall kind acts towards strangers, *F* (1715) = 249.95, *p* < 0.001. The interaction effect was significant, *F* (1715) = 12.05, *p* = 0.001, such that all participants recalled smaller kind acts for strangers than for close others, but this difference was greater when writing in Chinese than in English.

#### Main Results

Next, we tested our pre-registered hypothesis that the effect of the target of the recalled kindness depends on language. We predicted positive affect, negative affect, and affect valence (each with separate models) from target (dummy coded: stranger = 0, close other = 1), language (dummy coded: Chinese = 0, English= 1), order (effects coded: English-then-Chinese = − 0.5, Chinese-then-English = 0.5), and all possible interactions (i.e., three two-way interactions and one three-way interaction). This coding scheme allowed us to examine the target × language interaction controlling for (i.e., collapsing across) order and all possible interactions. We used multilevel modeling to account for the nesting of observations within people (i.e., because each participant completed measures twice, once in each language). These multilevel models had random intercepts, but not random slopes, which was necessary because each person had two observations. In our multilevel analyses, degrees of freedom were calculated with the Satterthwaite approximation.

**Fig. 1:**
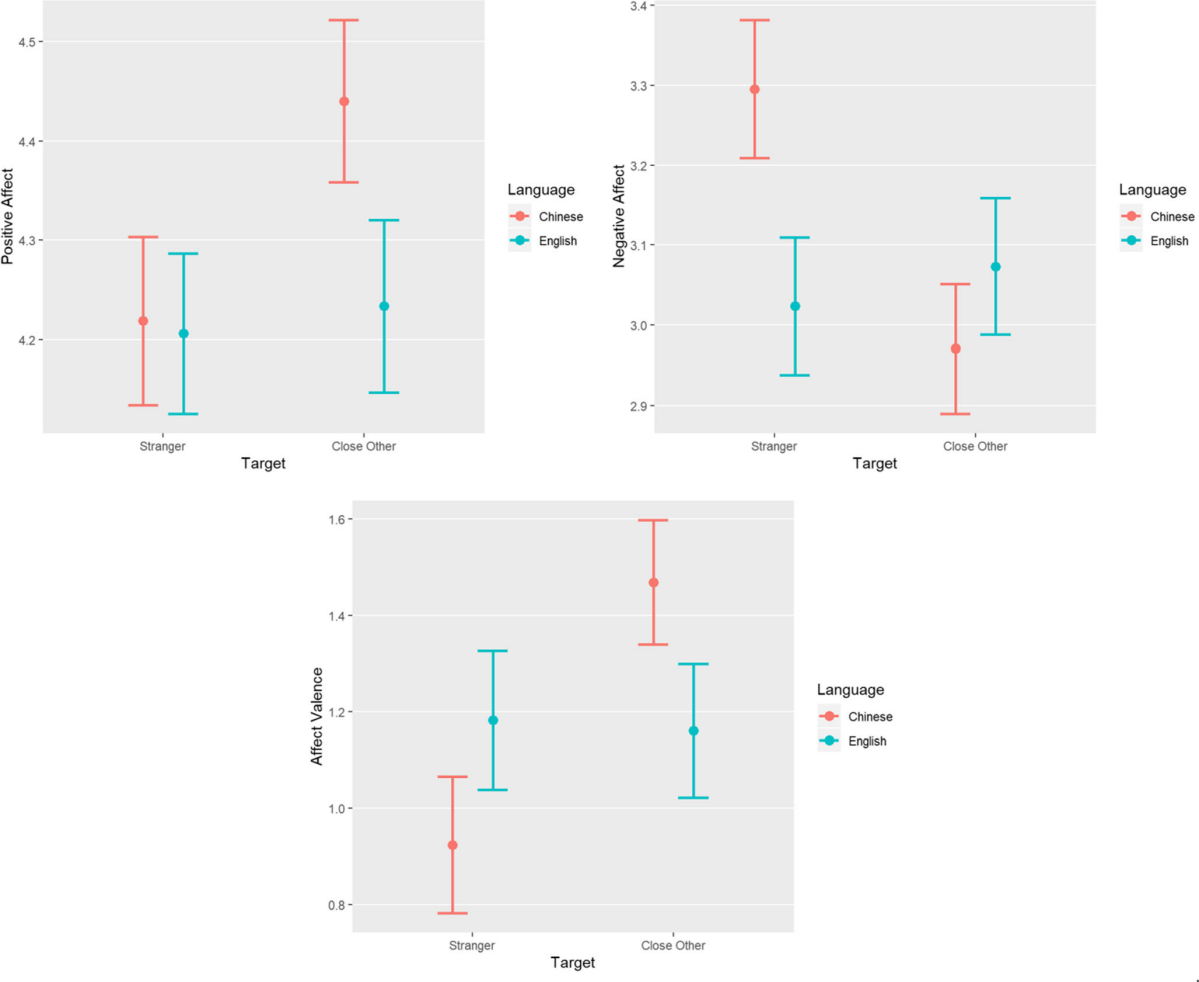
Positive affect, negative affect, and affect valence scores by target and language in Study 1

The results of these analyses showed that language significantly moderated the effect of target when predicting negative affect and affect valence, but not positive affect (see Table 1 and Fig. 1). However, the effect for positive affect was in the same direction as previous research (Shin, Walsh, & Lyubomirsky, 2019; Shin, Layous, Choi, Na, & Lyubomirsky, 2019) and marginally significant.

**Table 1:** Study 1: multilevel model results

Outcome	Predictor	b [95% CI]	Partial *r* [95% CI]	*p*
Positive Affect	Intercept	4.21 [4.05, 4.38]		< 0.001
	Language	− 0.02 [− 0.17, 0.13]	− 0.02 [− 0.12, 0.09]	0.770
	Order	0.30 [− 0.02, 0.63]	0.08 [− 0.01, 0.16]	0.068
	Target	0.23 [0.00, 0.45]	0.08 [0.00, 0.17]	0.051
	Language × order	0.04 [−0.26, 0.34]	0.01 [− 0.09, 0.12]	0.809
	Language × target	− 0.18 [− 0.38, 0.03]	− 0.09 [− 0.19, 0.02]	0.098
	Order × target	− 0.23 [− 0.69, 0.22]	− 0.04 [− 0.13, 0.04]	0.312
	Language × order × target	− 0.13 [− 0.54, 0.29]	− 0.03 [− 0.14, 0.07]	0.547
Negative Affect	Intercept	3.31 [3.14, 3.47]		< 0.001
	Language	− 0.27 [− 0.43, − 0.10]	− 0.17 [− 0.27, − 0.07]	0.001
	Order	− 0.24 [− 0.57, 0.09]	− 0.06 [− 0.14, 0.02]	0.158
	Target	− 0.33 [− 0.56, − 0.09]	− 0.12 [− 0.20, − 0.03]	0.006
	Language × order	0.26 [− 0.07, 0.58]	0.08 [− 0.02, 0.18]	0.127
	Language × target	0.35 [0.12, 0.57]	0.16 [0.06, 0.26]	0.003
	Order × target	0.14 [−0.32, 0.60]	0.03 [− 0.06, 0.11]	0.547
	Language × order × target	0.04 [−0.41, 0.50]	0.01 [− 0.10, 0.12]	0.849
Affect Valence	Intercept	0.91 [0.64, 1.18]		< 0.001
	Language	0.24 [.00, 0.48]	0.11 [0.00, 0.21]	0.048
	Order	0.54 [.00, 1.08]	0.09 [0.00, 0.17]	0.049
	Target	0.55 [0.17, 0.93]	0.12 [0.04, 0.21]	0.004
	Language × order	−0.22 [−0.70, 0.26]	− 0.05 [− 0.15, 0.06]	0.365
	Language × target	−0.51 [−0.84, −0.18]	− 0.16 [− 0.26, −0.06]	0.003
	Order × target	−0.37 [−1.12, 0.38]	− 0.04 [− 0.13, 0.04]	0.333
	Language × order × target	−0.17 [−0.83, 0.49]	− 0.03 [− 0.13, 0.08]	0.618

*Note.* A positive effect of language × target indicates that the degree to which those in the close others group scored higher than those in the stranger group was greater in English than Chinese

To explore the target × language interaction further, as pre-registered, we examined the simple effects of target within each language using two-sample *t* tests. Target significantly impacted negative affect and affect valence when participants completed the questionnaires in Chinese, such that negative affect was lower (mean difference = − 0.32, *r* = − 0.15, *p* = 0.01) and affect valence higher (mean difference = 0.55, *r* = 0.15, *p* = 0.00) for participants who wrote about kindness towards close others compared to strangers. These effects were not present when participants completed the questionnaires in English (mean difference for negative affect = 0.06, *r* = 0.03, *p* = 0.61; mean difference for affect valence = − 0.04, *r* = − 0.01, *p* = 0.85). A similar pattern emerged for positive affect, where target had a marginally significant impact in Chinese (with positive affect after recalling kindness towards close others higher than towards strangers; mean difference = 0.22, *r* = 0.10, *p* = 0.06) and a near-zero impact in English (mean difference = .02, *r* = .01, *p* = .85).^1^

### Discussion

Partially supporting our hypothesis, when participants were primed with the Chinese language (and thus, with their collectivist identity), they reported marginally higher positive affect and significantly higher affect valence—as well as significantly lower negative affect—when recalling kind acts towards close versus distant others. These results are consistent with the notion that members of Asian cultures are characterized by interdependent subjective well-being, in which harmonious relationships with those in one’s in-group are emphasized over those with one’s out-group (Hitokoto & Uchida, 2015; Triandis, 2001; Uchida et al., 2004).

When participants were primed in English (and thus, with their individualist identity), no differences in positive affect, negative affect, or affect valence were observed between those who recalled kindness towards close others and those who recalled kindness towards strangers. This pattern of results is in line with the concept of independent subjective well-being, in which the relationship with the target of one’s kind acts is less important than the kind act’s effect on one’s personal happiness through explicit striving (Uchida et al., 2004).

## Study 2

Study 2 aimed to test the effect of recalling a kindness that benefitted close others versus strangers in Asian (Hong Kong) versus European American individuals. To this end, we used a between-subjects design (comparing different cultures) rather than the within-subjects design (comparing priming of different identities) used in Study 1. This approach allowed us to obtain additional evidence that the effects found in Study 1 were driven by cultural differences and not differences in primary language fluency.

### Method

#### Design

This study used a 2 (target: stranger vs. close other) × 2 (culture: Hong Kong vs. USA) between-subjects factorial design. The final sample sizes for each condition were as follows: Stranger/Hong Kong (*n* = 48), Close Other/Hong Kong (*n* = 45), Stranger/USA (*n* = 54), and Close Other/USA (*n* = 52).

#### Participants

Undergraduate students from the USA (*n* = 106) and Hong Kong (*n* = 93) participated in this study. US participants were predominantly female (61.3%), with a mean age of 19.7 years (range = 18–24 years). They were 74.1% White, 12.3% Black, 7.1% Asian, 3.8% Hispanic, 2.4% more than one ethnicity, and 0.5% Native American. Hong Kong participants were also predominantly female (78.5%), with a mean age was 21.4 years (range = 18–27 years). They were 95% ethnically Chinese, 1.5% more than one ethnicity, 1% Singaporean, 1% Indian, 0.5% European, 0.5% Canadian, 0.5% Malaysian, and 0.5% Arab. Participants were eligible to join the study if they were able to read and write in English (in the USA) and in both English and Traditional Chinese (in Hong Kong, as some of the material was presented in both languages). US students received school credit in exchange for their participation, and Hong Kong students were compensated with $20 HKD.

#### Procedure

In the USA, students were recruited from the university’s participant pool, while in Hong Kong, students were recruited from a psychology course. The students logged-in to an online survey (hosted via Qualtrics.com) to receive instructions for a writing activity and complete measures. US participants were asked to recall and write about kind acts in English, and they completed a survey that was written entirely in English, including all instructions and measures. Hong Kong participants were asked to recall and write about kind acts in English as well, but they completed the main outcome measure written both in English and Chinese (Traditional or Simplified based on participants’ Chinese language preferences).

Prior to beginning the study, all participants were randomly assigned to one of two conditions that asked them to recall and write about a kind act they themselves had done towards (1) a stranger or (2) a close other. Next, participants were sent a survey link. After consenting, they completed demographic questions (e.g., age, sex). Then, participants completed their assigned recalling kindness writing activity (one of two writing prompts) and, afterwards, filled out the well-being outcome measure (i.e., positive and negative affect). Upon completing the survey, participants received a debriefing statement.

##### Recalling Kindness Writing Activity

Participants completed the exact same writing activity as in Study 1 about recalling kindnesses either towards a close other or stranger in either English or Chinese, depending on their condition assignment and culture.

#### Measures

For our US participants, all measures and instructions were written in English. For the Hong Kong participants, our primary outcome measure (the AAS) was translated into Chinese in advance by our collaborator in Hong Kong, and then checked and updated for accuracy by Chinese-English bilingual research assistants. Other measures (e.g., demographic) and instructions were written in English.

##### Affective Well-Being Outcome Measure

As in Study 1, we administered our 12-item modified AAS to assess the extent to which participants felt positive and negative emotions following the recalling kindness activity. Scale reliabilities (McDonald’s omegas) for positive affect, negative affect, and affect valence were 0.80, 0.73, and 0.80, respectively.

### Results

#### Manipulation Check

To check whether participants recalled comparable kind acts (vis-à-vis closeness to target and size of the act) in each culture, three raters independently coded the participants’ written descriptions of their recalled kind acts on two questions: “How close is the author to the person for whom they did an act of kindness? (1, not at all; 4, moderately; 7, very) and “How large was the task?” (1, extremely small [could do the task effortlessly or with little attention (e.g., holding door open for someone)]; 4, moderately [required a little more effort and/or attention (e.g., giving up a subway seat to a pregnant woman)]; 7, extremely large [could only do the task with effort and attention to the task at that moment (e.g., changing a tire)]).

Intraclass correlations for these two questions were excellent, at 0.94 and 0.76, respectively. Both coding questions were analyzed with a 2 (Target: Stranger vs. Close Other) × 2 (Culture: Hong Kong vs. USA) ANOVA. Mean values for the closeness-to-target coding were as follows: Stranger-Hong Kong *M* = 2.31, Close Other-Hong Kong *M* = 5.51, Stranger-USA *M* = 1.92, and Close Other-USA *M* = 5.70. Mean values for the size-of-act coding were Stranger-Hong Kong *M* = 3.26, Close Other-Hong Kong *M* = 4.30, Stranger-USA *M* = 3.48, and Close Other-USA *M* = 4.92.

With regard to the closeness coding, those who were asked to recall kind acts towards close others were coded as reporting kind acts towards people closer to them than those who were asked to recall kind acts towards strangers, *F* (1194) = 273.58, *p* < 0.001. Notably, however, neither the main effect of culture, *F* (1194) = 0.24, *p* = 0.63, nor the interaction, *F* (1194) = 1.80, *p* = 0.18, were significant. That is, the US and Hong Kong participants did not differ in how close a target they chose to recall their kind acts (in either the stranger or close other conditions). Furthermore, Hong Kong participants did not write about targets closer to them in the close other (versus distant other) condition than did US participants.

Regarding codings of the size of the kindness, US participants reported bigger acts than Hong Kong participants, *F* (1195) = 4.10, *p* = 0.04. Additionally, not surprisingly, our codings revealed that those who were asked to recall kind acts towards close others recalled bigger acts than those who were asked to recall kind acts towards strangers, *F* (1195) = 36.74, *p* < 0.001. Finally, the interaction effect was not significant, *F* (1195) = 0.99, *p* = 0.32, suggesting that the sizes of kindnesses described were no more similar for strangers and close others among US than Hong Kong participants.

#### Main Results

**Fig. 2:**
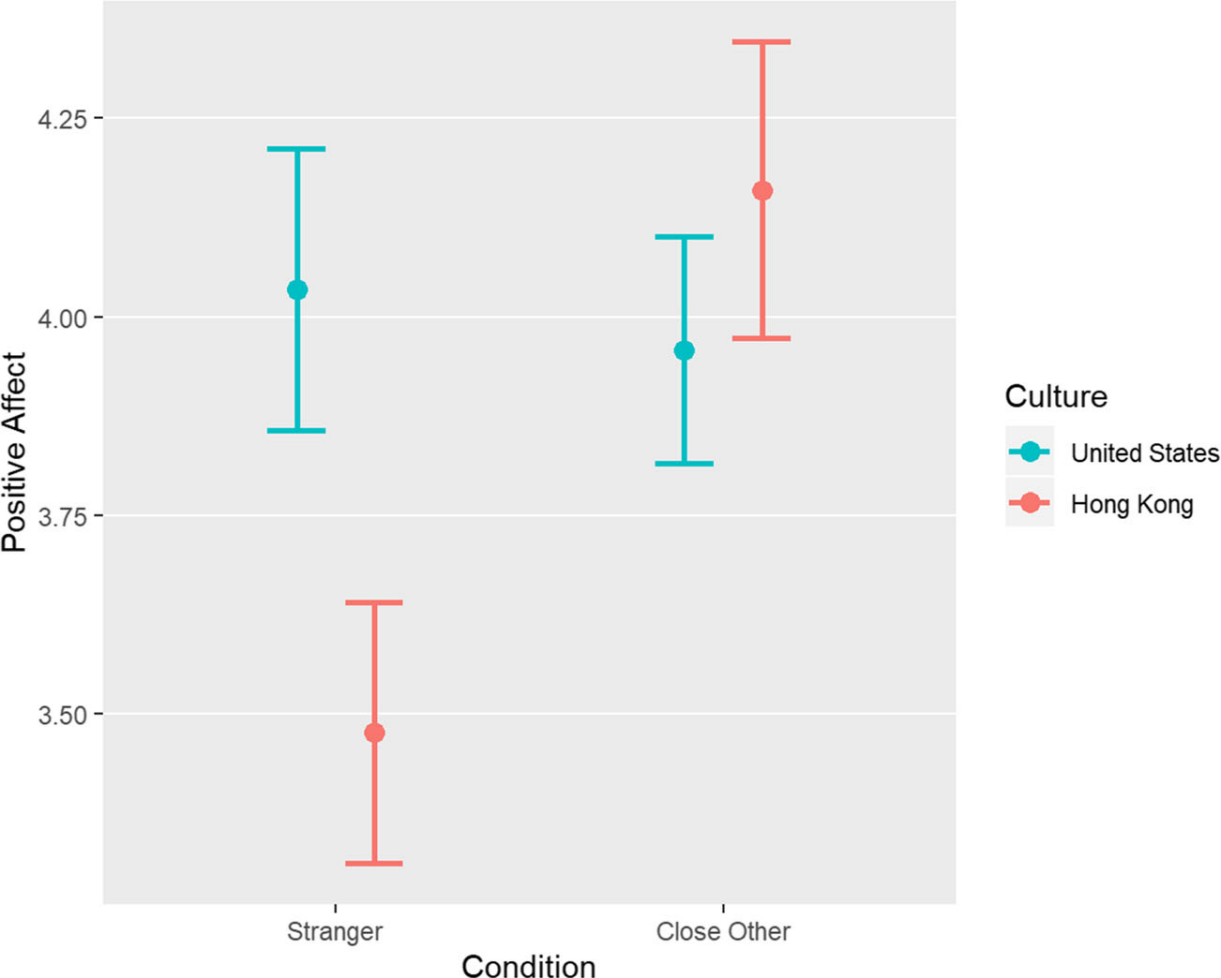
Positive affect scores by target and culture in Study 2

First, we tested our hypothesis that the effect of the target of the recalled acts of kindness depends on culture. To this end, we predicted positive affect, negative affect, and affect valence (each with separate regression models) from target (dummy coded: stranger = 0, close other = 1), culture (dummy coded: Hong Kong = 0, USA = 1), and their interaction. The target × culture interaction term was only significant when predicting positive affect, b = − 0.76, partial *r* = − 0.16, *p* = 0.03 (see Fig. 2).

To unpack the target × culture interaction further, we conducted two-sample *t* tests to assess simple effects. In each culture, we predicted each outcome from target. As illustrated in Fig. 2, target significantly impacted positive affect (mean difference = 0.68, *r* = 0.28, *p* = 0.01) and marginally significantly impacted affect valence in Hong Kong (mean difference = 0.31, *r* = 0.20, *p* = 0.06; with close other greater than stranger) but not in the USA (mean difference for positive affect = − 0.08, *r* = − 0.03, *p* = 0.74; mean difference for affect valence = 0.01, *r* = 0.01, *p* = 0.95). No other simple effects were significant (mean difference for negative affect in Hong Kong = − 0.06, *r* = − 0.04, *p* = 0.72; mean difference for negative affect in USA: = 0.09, *r* = 0.05, *p* = 0.59).

### Discussion

As predicted and similar to Study 1, respondents from Hong Kong who recalled kind acts towards close others showed higher positive affect and marginally greater affect valence than those who recalled kind acts towards strangers. It is unclear why we did not observe differences in negative affect in the Hong Kong sample between those who recalled kind acts towards strangers and those who recalled kind acts towards close others. Notably, however, the US sample who recalled kind acts towards close others, as opposed to strangers, showed neither differences in positive affect, negative affect, nor affect valence.

## General Discussion

In two studies, collectivist culture-primed or collectivist participants reported more positive emotional experience when recalling kind acts towards friends and family than recalling kind acts towards strangers. These results provide evidence for interdependent subjective well-being in collectivist cultures, which places priority on close in-group relationships relative to out-group relationships (Hitokoto & Uchida, 2015; Triandis, 2001; Uchida et al., 2004). By contrast, individualist culture-primed or individualist participants did not experience differences in emotion when recalling kind acts towards friends and family and when recalling kind acts towards strangers. These results are consistent with the notion of independent subjective well-being (Uchida et al., 2004). The significance of these findings is underscored by the fact that we found parallel results using two different paradigms—comparing Eastern/Western bicultural individuals with a language priming paradigm and comparing members of Eastern and Western cultures directly. Importantly, by using participants from two different cultures with different first languages, Study 2 ruled out the possibility that the reason that recalling kind acts towards close others was more beneficial for Hong Kong bilinguals in Study 1 was that they were relatively more likely to have Chinese as their first/primary language.

Coding the kind acts in Study 1 on two different dimensions helped to illuminate these findings. Analyses of the closeness coding revealed two main effects and no interaction effect—namely, that recalling in English and recalling kindnesses towards close others led people to write about people relatively closer to them. These findings are inconsistent with the idea that our participants reported higher well-being when recalling kindness towards close others in Chinese simply because they chose targets to whom they felt closer when recalling in Chinese. We also coded the size of the kind acts that participants recalled and found that recalling in English led people to report bigger kindnesses overall than when recalling in Chinese. Notably, the interaction effect was significant, such that participants recalled smaller acts towards strangers (relative to close others) when writing in Chinese than when writing in English. These findings suggest a key mechanism for why people show relatively lower well-being when recalling kind acts towards strangers in Chinese than in English—namely, because they are recalling relatively smaller kind acts (e.g., helping a stranger with directions versus volunteering at a home for the elderly). In Study 2, analogous codings revealed no interaction effects, suggesting that our findings of cross-cultural differences are not likely to be accounted for by participants from Hong Kong recalling different types of kind acts towards close others versus strangers.

Importantly, our findings indicate that individuals who are bicultural may benefit from either interdependent-type or independent-type positive activities (e.g., recalling kindness to close others vs. strangers), depending on which of their cultural identities is made salient via language priming. These findings hold potential value to well-being scientists and cross-cultural investigators, as well as to practitioners, for how to cultivate happiness in individuals with individualist versus collectivist identities. Our results are consistent with the idea that the well-being of one’s in-group is more critical for members of collectivist than individualist cultures. As such, positive activity interventions implemented among people with collectivist identities may be more successful if they focus on strengthening connections with in-group members, such as with significant others, close friends, and family members.

### Limitations and Future Directions

A limitation of our studies worth noting is their reliance on undergraduate student samples; hence, our results are restricted to a particular age group, socioeconomic class, and education level. Future investigations are needed to determine whether our findings will replicate in older, working community samples. Moreover, because our work focused on two specific collectivist and individualist cultures—namely, Hong Kong and the USA—we cannot confidently generalize to other cultures that are considered collectivist (e.g., India) and individualist (e.g., Australia).

Furthermore, a neutral control group—no-kindness (e.g., “recall your breakfast”) or no-treatment (e.g., “recall nothing”)—would have illuminated whether the affective differences we observed reflected *boosts*, *flatlines*, or *declines*. This approach—feasible when there is enough power to include additional conditions—would allow investigators to answer questions about the direction of hedonic shifts (e.g., is one’s affect boosted or is it buffered against a downturn?) and is an important aim for future studies. However, we believe that our key finding is that collectivist cultures but not individualist ones (whether primed by language or residency) showed *different* levels of hedonic well-being in response to our manipulation.

Notably, both studies assessed affect immediately after participants were administered the recalling kindness PAIs. As such, these studies provide initial evidence for the short-term hedonic benefits of our interventions. Future studies should explore the long-term durability of these effects. However, even short-term boosts in hedonic well-being can be useful to practitioners and laypeople. Small effects (if repeated over time) can aggregate to produce meaningful changes in targeted outcomes (Funder & Ozer, 2019).

Future investigators could also test whether our recommendation vis-à-vis the primacy of close ties in people with collectivist identities applies to interventions that call for actually engaging in (versus simply recalling) acts of kindness or prosocial spending (Chancellor et al., 2018; Dunn et al., 2008; Nelson et al., 2016), as well as to writing letters of gratitude (Seligman et al., 2005; Layous et al., 2017; but see Layous, Nelson, & Lyubomirsky, 2013; Layous, Lee, Choi, & Lyubomirsky, 2013) or practicing loving-kindness meditation (Fredrickson et al., 2008). Notably, all of these types of positive activities involve directing attention away from oneself and onto other people in one’s life, a hallmark of collectivist cultures (Hitokoto & Uchida, 2015; Uchida et al., 2004). Future research, however, could explore whether a potential downside of concentrating on close ties involves missing out on opportunities to connect with strangers, even during brief interactions (see Epley & Schroeder, 2014; Sandstrom & Dunn, 2014, for well-being benefits in individualist cultures).

### Concluding Words

Although many collectivist cultures report lower happiness than individualist ones (Diener et al., 1995), researchers have not prioritized developing positive activities that address the unique characteristics of these cultures. We believe that our studies contribute to understanding how to better tailor positive activities to maximize their potential in increasing positive emotions in residents of Hong Kong and perhaps in members of other Asian and collectivist countries. Specifically, we showed that positive (i.e., prosocial) activities in such cultures promote emotional well-being when they focus on relationships with close as opposed to distant others. Furthermore, we demonstrated the feasibility and efficacy of using a language priming paradigm to test hypotheses about cultural differences in positive activities. The hope is that, through these and other empirical approaches, well-being science directs its attention to maximizing the emotional experience of not only Americans and Europeans but members of every culture around the world.

## Supplementary Information

ESM 1 (DOCX 35 kb)

## Data Availability

Data, methods, and materials for Study 1 were pre-registered on the Open Science Framework and can be found at https://tinyurl.com/y9ld6du4

## Footnotes

1 We also examined whether any potential moderators interacted with the target × language effect. The list of moderators and results are located in Supplemental Material.
