# Supplementary material for: Cultural Differences in the Hedonic Rewards of Recalling Kindness: Priming Cultural Identity with Language
Source: Affect Sci. 2021 Mar 23;2(1):80–90. doi: 10.1007/s42761-020-00029-3 (PMC9382926; doi:10.1007/s42761-020-00029-3)
Supplement: Supplementary file 1 — (DOCX 35 kb) [file 42761_2020_29_MOESM1_ESM.docx]

**Supplemental Material**

Below is a list of the moderators and outcomes that were measured but not presented in the main manuscript.

**Study 1 Moderators**

The following moderator measures were administered at the beginning of both sessions (T_1_ and T_2_) in the following order.

**Subjective Socioeconomic Status (SES).** We measured subjective SES with the MacArthur Scale of Subjective Social Status (Adler, Epel, Castellazzo, & Ichovics, 2000). This single-item measure asks participants to place themselves on a pictured ladder (with hypothetical rungs 1 through 10), such that those at the top of the ladder (10) are the best off and those at the bottom of the ladder (1) are the worst off in terms of SES-related outcomes, such as money and education.

**Personality.** We assessed participants’ trait-level extraversion, agreeableness, conscientiousness, negative emotionality, and open-mindedness with the Big Five Inventory (BFI; John & Srivastava, 1999). Participants responded to 21 items (e.g., “I am someone who … tends to be disorganized”) on a 5-point Likert scale (1 = *disagree strongly*, 5 = *agree strongly*). The reliabilities (McDonald’s omegas [ωs]) at T_1_ and T_2_, respectively, were .80 and .79 for extraversion, .54 and .55 for agreeableness, .59 and .60 for conscientiousness, .64 and .67 for negative emotionality, and .49 and .37 for open-mindedness.

**Cultural Identity.** We used several different scales to measure cultural identity at both time points. First, participants responded to three subjective cultural identity questions: “From 0 to 100 percent, to what extent do you feel … “Eastern/Asian/Chinese,” “Western/European/American,” and “Like you belong to another cultural group that is not Eastern/Asian/Chinese or Western/European/American.” If these three percentages did not sum to 100 for a particular individual, we rescored them so that they did sum to 100 while preserving their relative proportions. Then, we created a composite (“culture percent”) by subtracting the “Eastern/Asian/Chinese” percentage from the “Western/European/American” percentage (ωs = .82, .85).

A second set of questions were adapted from the Suinn-Lew Asian Self-Identity Acculturation Scale (Suinn, Rickard-Figueroa, Lew, & Vigil, 1987), and asked participants to rate their identity and values with 5 items on 7-point Likert scales (1 = *Completely Eastern/Asian/Chinese*, 7 = *Completely Western/European/American*). A composite of these scales (“subjective culture”) had ωs of .53 and .43.

As a final measure of cultural identity, we administered a 16-item scale assessing vertical and horizontal individualism and collectivism (IND-COL; Triandis & Gelfand, 1998). Example items include “I’d rather depend on myself than others” (horizontal individualism; ωs = .83, .82), “It is important for me to do my job better than others (vertical individualism; ωs = .73, .74), “If a co-worker gets a prize, I would feel proud” (horizontal collectivism; ωs = .68, .77), and “It is my duty to take care of my family, even when I have to sacrifice what I want (vertical collectivism; ωs = .75, .81). Participants were asked to rate their level of agreement with these items on a 7-point Likert scale (1 = *strongly disagree*, 7 = *strongly agree*).

**Study 1 Moderator Results**

We examined whether any of our potential moderators interacted with the target $\times$ language effect. For each outcome and each moderator, we conducted a multilevel model with target, language, and order and a potential moderator with all possible interaction terms. We examined the target $\times$ language $\times$ potential moderator interaction effect in each model; however, none were significant.

*Moderators of the Target* $\times$ *Language Effect*

| Outcome | Moderator | b [95% CI] | Partial *r* [95% CI] | *t* | df | *p* |
| --- | --- | --- | --- | --- | --- | --- |
| Positive Affect | Order | -0.12 [-0.54, 0.29] | -.03 [-.14, .07] | -0.58 | 344.83 | .56 |
| Positive Affect | Age | 0.00 [-0.05, 0.05] | .00 [-.11, .11] | -0.02 | 335.90 | .98 |
| Positive Affect | Sex | -0.13 [-0.65, 0.38] | -.03 [-.13, .08] | -0.51 | 347.66 | .61 |
| Positive Affect | Parents’ Education | 0.15 [-0.04, 0.34] | .08 [-.02, .18] | 1.51 | 354.21 | .13 |
| Positive Affect | MacArthur SES | 0.12 [-0.04, 0.27] | .08 [-.03, .18] | 1.47 | 360.83 | .14 |
| Positive Affect | Extraversion | 0.01 [-0.26, 0.27] | .00 [-.10, .10] | 0.04 | 376.90 | .97 |
| Positive Affect | Negative Emotionality | -0.05 [-0.31, 0.21] | -.02 [-.12, .08] | -0.36 | 375.62 | .72 |
| Positive Affect | Conscientiousness | 0.02 [-0.29, 0.33] | .01 [-.09, .11] | 0.13 | 381.41 | .90 |
| Positive Affect | Agreeableness | 0.06 [-0.35, 0.47] | .01 [-.08, .11] | 0.28 | 411.39 | .78 |
| Positive Affect | Open Mindedness | 0.03 [-0.23, 0.29] | .01 [-.09, .11] | 0.24 | 392.08 | .81 |
| Positive Affect | Subjective Culture | -0.06 [-0.46, 0.34] | -.01 [-.11, .08] | -0.29 | 388.73 | .77 |
| Positive Affect | Culture Percent | 0.00 [-0.01, 0.01] | .00 [-.11, .10] | -0.08 | 376.49 | .93 |
| Positive Affect | Individualism Horizontal | -0.08 [-0.32, 0.17] | -.03 [-.13, .07] | -0.62 | 382.71 | .54 |
| Positive Affect | Individualism Vertical | 0.13 [-0.10, 0.36] | .06 [-.04, .16] | 1.12 | 373.80 | .26 |
| Positive Affect | Collectivism Horizontal | 0.07 [-0.23, 0.36] | .02 [-.08, .12] | 0.44 | 393.79 | .66 |
| Positive Affect | Collectivism Vertical | -0.02 [-0.26, 0.22] | -.01 [-.11, .09] | -0.18 | 381.23 | .86 |
| Positive Affect | Birthplace | -0.22 [-0.74, 0.3] | -.05 [-.15, .06] | -0.84 | 342.53 | .40 |
| Positive Affect | Speak English | 0.08 [-0.36, 0.52] | .02 [-.08, .12] | 0.36 | 377.11 | .72 |
| Positive Affect | Speak Cantonese | 0.30 [-0.86, 1.46] | .03 [-.08, .13] | 0.51 | 337.26 | .61 |
| Positive Affect | Speak Mandarin | 0.10 [-1.22, 1.41] | .01 [-.09, .11] | 0.15 | 377.68 | .88 |
| Positive Affect | Speak Other | 0.12 [-0.31, 0.55] | .03 [-.07, .13] | 0.55 | 369.97 | .58 |
| Negative Affect | Order | 0.03 [-0.42, 0.49] | .01 [-.10, .11] | 0.14 | 341.71 | .89 |
| Negative Affect | Age | 0.01 [-0.05, 0.07] | .01 [-.09, .12] | 0.26 | 334.99 | .79 |
| Negative Affect | Sex | -0.28 [-0.85, 0.3] | -.05 [-.15, .05] | -0.95 | 346.97 | .34 |
| Negative Affect | Parents’ Education | -0.03 [-0.24, 0.17] | -.02 [-.12, .09] | -0.32 | 350.96 | .75 |
| Negative Affect | MacArthur SES | 0.02 [-0.15, 0.19] | .01 [-.09, .12] | 0.25 | 366.32 | .80 |
| Negative Affect | Extraversion | -0.16 [-0.45, 0.13] | -.06 [-.16, .05] | -1.07 | 374.63 | .28 |
| Negative Affect | Negative Emotionality | 0.07 [-0.21, 0.36] | .03 [-.07, .12] | 0.51 | 389.94 | .61 |
| Negative Affect | Conscientiousness | -0.09 [-0.42, 0.25] | -.03 [-.12, .07] | -0.50 | 388.86 | .61 |
| Negative Affect | Agreeableness | 0.17 [-0.27, 0.61] | .04 [-.06, .13] | 0.76 | 417.44 | .45 |
| Negative Affect | Open Mindedness | -0.23 [-0.51, 0.05] | -.08 [-.18, .02] | -1.60 | 396.81 | .11 |
| Negative Affect | Subjective Culture | -0.05 [-0.48, 0.39] | -.01 [-.11, .09] | -0.21 | 391.90 | .83 |
| Negative Affect | Culture Percent | 0.00 [-0.02, 0.01] | -.02 [-.12, .08] | -0.44 | 382.20 | .66 |
| Negative Affect | Individualism Horizontal | -0.04 [-0.31, 0.22] | -.02 [-.12, .08] | -0.32 | 386.99 | .75 |
| Negative Affect | Individualism Vertical | -0.03 [-0.29, 0.22] | -.01 [-.11, .09] | -0.26 | 379.32 | .80 |
| Negative Affect | Collectivism Horizontal | 0.01 [-0.32, 0.33] | .00 [-.10, .10] | 0.03 | 403.14 | .97 |
| Negative Affect | Collectivism Vertical | 0.11 [-0.15, 0.37] | .04 [-.06, .14] | 0.84 | 383.42 | .40 |
| Negative Affect | Birthplace | 0.54 [-0.03, 1.11] | .10 [-.01, .20] | 1.87 | 339.25 | .06 |
| Negative Affect | Speak English | -0.05 [-0.52, 0.43] | -.01 [-.11, .09] | -0.21 | 379.08 | .84 |
| Negative Affect | Speak Cantonese | 0.30 [-0.97, 1.58] | .03 [-.08, .13] | 0.47 | 333.44 | .64 |
| Negative Affect | Speak Mandarin | -0.31 [-1.74, 1.11] | -.02 [-.12, .08] | -0.43 | 380.44 | .67 |
| Negative Affect | Speak Other | 0.00 [-0.47, 0.48] | .00 [-.10, .10] | 0.02 | 375.69 | .99 |
| Affect Valence | Order | -1.20 [-5.47, 3.07] | -.03 [-.13, .07] | -0.55 | 381.92 | .58 |
| Affect Valence | Age | -0.01 [-0.09, 0.07] | -.01 [-.12, .10] | -0.21 | 335.30 | .84 |
| Affect Valence | Sex | 0.15 [-0.68, 0.99] | .02 [-.09, .12] | 0.36 | 346.55 | .72 |
| Affect Valence | Parents’ Education | 0.18 [-0.12, 0.48] | .06 [-.04, .17] | 1.17 | 350.26 | .24 |
| Affect Valence | MacArthur SES | 0.09 [-0.16, 0.34] | .04 [-.07, .14] | 0.71 | 358.97 | .48 |
| Affect Valence | Extraversion | 0.16 [-0.26, 0.58] | .04 [-.06, .14] | 0.74 | 372.50 | .46 |
| Affect Valence | Negative Emotionality | -0.11 [-0.54, 0.31] | -.03 [-.13, .07] | -0.53 | 378.22 | .60 |
| Affect Valence | Conscientiousness | 0.13 [-0.37, 0.62] | .03 [-.08, .13] | 0.51 | 376.56 | .61 |
| Affect Valence | Agreeableness | -0.10 [-0.75, 0.56] | -.01 [-.11, .08] | -0.30 | 400.21 | .77 |
| Affect Valence | Open Mindedness | 0.27 [-0.15, 0.69] | .06 [-.04, .16] | 1.27 | 384.90 | .20 |
| Affect Valence | Subjective Culture | -0.01 [-0.65, 0.63] | .00 [-.10, .10] | -0.03 | 382.27 | .98 |
| Affect Valence | Culture Percent | 0.00 [-0.02, 0.02] | .01 [-.09, .11] | 0.20 | 373.54 | .84 |
| Affect Valence | Individualism Horizontal | -0.03 [-0.43, 0.36] | -.01 [-.11, .09] | -0.16 | 375.08 | .87 |
| Affect Valence | Individualism Vertical | 0.17 [-0.20, 0.54] | .05 [-.06, .15] | 0.89 | 368.97 | .37 |
| Affect Valence | Collectivism Horizontal | 0.07 [-0.40, 0.55] | .02 [-.08, .11] | 0.30 | 388.23 | .77 |
| Affect Valence | Collectivism Vertical | -0.12 [-0.50, 0.26] | -.03 [-.13, .07] | -0.63 | 373.10 | .53 |
| Affect Valence | Birthplace | -0.77 [-1.60, 0.07] | -.10 [-.20, .01] | -1.81 | 339.41 | .07 |
| Affect Valence | Speak English | 0.12 [-0.57, 0.81] | .02 [-.08, .12] | 0.34 | 369.15 | .73 |
| Affect Valence | Speak Cantonese | -0.02 [-1.87, 1.83] | .00 [-.11, .11] | -0.02 | 335.23 | .99 |
| Affect Valence | Speak Mandarin | 0.46 [-1.63, 2.54] | .02 [-.08, .12] | 0.43 | 370.80 | .67 |
| Affect Valence | Speak Other | 0.09 [-0.60, 0.77] | .01 [-.09, .12] | 0.25 | 364.75 | .80 |

*Note.* Positive effects indicate that higher scores on the moderator were associated with a larger Target $\times$ Language Effect

**Additional Measures Administered in Study 1**

**Brief Well-Being Indicators** (Jacobs Bao, 2012)

**Balanced Measure of Psychological Needs** (Sheldon, Elliot, Kim, & Kasser, 2001)

**Brief State Humility Scale** (Kruse, Chancellor, & Lyubomirsky, 2017)

**Supplemental Material References**

Adler, N. E., Epel, E. S., Castellazzo, G., & Ickovics, J. R. (2000). Relationship of subjective and objective social status with psychological and physiological functioning: Preliminary data in healthy, White women. *Health Psychology, 19*(6), 586-592.

Jacobs Bao, K. (2012). The course of well-being in Romantic Relationships: Predicting positive affect in dating participants. *Psychology, 3*(12A), 1091–1099. doi: 10.4236/psych.2012.312A161

John, O. P., & Srivastava, S. (1999). The Big Five trait taxonomy: History, measurement, and theoretical perspectives. In L. A. Pervin, & O. P. John (Eds.), *Handbook of personality: Theory and research* (2nd ed., pp. 102-138). New York: Guilford.

Kruse, E., Chancellor, J., & Lyubomirsky, S. (2017). State humility: Measurement, conceptual validation, and intrapersonal processes. *Self and Identity, 16,*399-438. doi: 10.1080/15298868.2016.1267662

Sheldon, K. M., Elliot, A. J., Kim, Y., & Kasser, T. (2001). What is satisfying about satisfying events? Testing 10 candidate psychological needs. *Journal of Personality and Social Psychology, 80*(2), 325–339. doi: 10.1037/0022-3514.80.2.325

Suinn, R. M., Rickard-Figueroa, K., Lew, S., & Vigil, P. (1987). The Suinn-Lew Asian Self-Identity Acculturation Scale: An initial report. *Educational and Psychological Measurement, 47*(2), 401-407.

Triandis, H. C., & Gelfand, M. J. (1998). Converging measurement of horizontal and vertical individualism and collectivism. *Journal of Personality and Social Psychology, 74,* 118-128.
